# Recovery journey of people with a lived experience of schizophrenia: a qualitative study of experiences

**DOI:** 10.1186/s12888-023-04862-1

**Published:** 2023-06-27

**Authors:** Min Ma, Zhidao Shi, Yanhong Chen, Xiquan Ma

**Affiliations:** 1 Wuhan Mental Health center, Wuhan Hospital for Psychotherapy, Rehabilitation Department, Wuhan, China; 2 Clinical Research Center for Mental Disorders, Shanghai Pudong New Area Mental Health Center, School of Medicine, Tongji University, Shanghai, China; 3 China University of Geosciences Wuhan, Wuhan, China; 4 Shanghai Children’s Medical Center, School of Medicine, Shanghai Jiao Tong University, Shanghai, China

**Keywords:** Schizophrenia, Recovery, Qualitative research, Family, Culture

## Abstract

**Background:**

Mental health recovery involves an integration of clinical and psychosocial frameworks. The recovery journey of individuals diagnosed with schizophrenia and the factors that influence it have been extensively studied. Because the recovery journey is culturally influenced, we examined the recovery process expriences of individuals diagnosed with schizophrenia in China, focusing on the influence of a Confucian-dominated collectivist and family-centred culture.

**Methods:**

An Interpretive Phenomenological Analysis (IPA) study was conducted; data were gathered through in-depth interviews with 11 individuals with lived experience of schizophrenia.

**Results:**

Four themes were identified in this study: traumatic illness experiences, influence of the family, motives for recovery, and posttraumatic growth, comprising ten subthemes. “For the family” and “relying on oneself” are the main drivers of recovery for individuals with a Chinese cultural background. Some people believe that taking care of themselves is an important way to ease the burden on their families and treat them well. There is a link between ‘for the family’ and ‘relying on oneself.

**Conclusions:**

Individuals living with schizophrenia in China have undergone significant traumatic experiences and have profound interactions with their families. Post-traumatic growth reflects an increase in the individual’s connection to others and individual agency. It also suggests that the individual is not receiving enough support outside of the family. The impact of individual agency and family relationships should be considered in services that promote recovery, and clinic staff should enhance support outside the home to the individuals.

**Supplementary Information:**

The online version contains supplementary material available at 10.1186/s12888-023-04862-1.

## Background

Schizophrenia is a severe psychiatric disease, and it usually causes social dysfunction in individuals [1]. In 2017, 19.78 million individuals were diagnosed with schizophrenia worldwide, a 62.74% increase from 1990 [2]. In a 2022 report prompted, although the crude incidence of schizophrenia has decreased in China, the age-standardized incidence rate (ASIR), ,crude disability-adjusted life year (DALY) rate and age-standardized DALYs rate (ASDR)all showed a generally increasing trend over the last three decades [3]. In China, schizophrenia has become an important public health problem [4].

With the rise of the recovery movement, individuals are increasingly writing about their treatment experiences and journey to recovery, raising awareness that recovery from schizophrenia is possible [5–8]. The results reported in these studies indicate that approxmately half of the participants recover or significantly improve over the long term, suggesting that remission or recovery is much more common than previously thought [9, 10]. On the other hand, by studying the stories of those who have recovered, it is possible to tease out what recovery means to them, what factors affected and helped their recovery and what they think is critical to the recovery process [11].

Spaniol and his colleagues pointed out that the four broad stages of mental illness recovery are being overwhelmed by the illness, fighting with the illness, coexisting with the illness, and surpassing the illness. The three main steps related to the recovery process are (1) an interpretive framework for understanding the experience of severe mental problems (2) gradually controlling mental illness and (3) obtaining a meaningful, productive, and valuable status in society. For individuals, understanding the experience of serious mental problems is their first step towards recovery [12].

Brammer believes that recovery is neither a matter of biomedical/clinical nor psychosocial recovery; it is an integration of clinical and psychosocial frameworks [13].

Sambeek and colleagues point out that researchers often ignore the sociocultural context of the narrative [14] or focus only on its personal or social dimensions [15].

Some cross-cultural studies suggest that cultural differences can lead to differences in individuals’ stigmatizing attitudes towards mental illness [16, 17] and can also influence the psychological experience of family members in caring for individuals and how they care for them [18, 19].

Chinese culture is dominated by collectivism under Confucianism. In Chinese culture, family and individual bonds are solid and interdependent [20]. In this context, does the recovery process differ from that in the West?

Some researchers have explored the field, and Yen-Ching Chang has highlighted the influence of Chinese culture on recovery-oriented services. He identified the search for cooperation from family members and the elimination of stigmatizing influences as the main challenges faced by professionals in a non-Western context [21].

Eva Yin-han Chung’s review of several papers argues that the concepts and philosophies of Western community-based rehabilitation cannot be directly applied to the Chinese context. Chinese cultural values have influenced CBR practice in Chinese communities [22]. Traditional peasant culture, traditional Chinese philosophy, and socialist ideology primarily influence current rehabilitation and CBR practices in China. Traditional cultural beliefs influence community members’ views of health, disability, autonomy, and family relationships [23]. For this reason, many argue that introducing externally planned CBR programs is counterproductive because they need to consider local needs and existing local practices. Therefore Eva Yin-han Chung claims an appropriate model or framework is needed to adapt to the unique Chinese cultural context and to guide practice in the Chinese community [22].

The above research suggests that the theory of recovery in China needs to consider Chinese culture. Researching Chinese people with lived experiences of schizophrenia recovery can help us understand the recovery process of individuals and consider the similarities and differences in the recovery journey of people with lived experiences of schizophrenia acoss cultures.

## Participants and methods

### Participants

Participants were recruited through clinical staff at the Wuhan Mental Health Centre from November 2017 - March 2018. Participation was voluntary and was possible only with informed consent. The Inclusion criteria were (1) being diagnosed with schizophrenia according to the International Statistical Classification of Diseases and Related Health Problems “Diagnostic criteria for schizophrenia in the 10th edition; (2) having experienced at least two relapses or having residual symptoms but a current BPRS score of less than 35 on the 18-item BPRS as scored by the psychiatrist responsible for recruitment. [12](The verbal expression of individual with the severe condition are impacted. Therefore we used the BPRS as a screening tool. We wanted the participants to be able to express themselves well enough to articulate the themes we wanted to explore. ); (3) recieving a participant information sheets from staff, from which potential participants could ask questions of the staff; and (4) being wlling to participate in the qualitative research interview and signing an informed consent form after reading the informed consent form. The exclusion criteria were (1) having an intellectual disability; (2) having severe physical or cerebral organic diseases; (3) abusing or being dependent on psychoactive substance. Eleven individuals were finally enrolled.

The study’s sample selection mainly used the purposive sampling method and followed the principle of saturation. The interviews are conducted face-to-face; each lastiong from 1 to 1.5 h. The interview location was in the psychotherapy room of the hospital. After obtaining the individual’s consent, signed the informed consent form, the interview was performed and recorded. The research team consisted of three psychiatrists and a graduate student in psychology. The interviewer was a graduate student in psychology. All of the researchers were trained in and had previously conducted qualitative research. Some individuals underwent supplementary interviews according to the needs of the investigator. We used a code assigned to each participant to ensure anonymity.

### Procedure

We adopt the interpretative phenomenological analysis (IPA) method in this study. IPA was developed by Jonathan A. As a qualitative research method in the fields of health psychology and social science, IPA focuses on how people perceive experience, that is, it studies their experience living in the world [24, 25]. The hypothesis of IPA is that the content of the participant’s psychological world that the analyst pays attention to may be manifested in the form of belief and structured by the participant’s words, or the participant’s story itself represents the identity of the participant [25, 26]. IPA researchers want to analyse in detail how participants perceive and attach meaning to events that happen to them, so they need a flexible means of data collection. IPA mainly uses semistructured interviews to collect data [26, 27].

This study aimed at exploring the illness-related experiences and recovery processes of people who have experienced schizophrenia. The interviews mainly focused on how they get sick, how they think about the illness and the impact of the illness on themselves. How do they cope with these effects? How is recovery perceived, what is good for recovery, and what is bad for recovery. We also asked individuals to report their current living conditions. (An outline of the interview is available within the Supplementary Material) The interview was semistructured and interactive. The researcher asked open questions and clarified the answers encouraged the individual to express themselves as completely as possible until they felt there was nothing more to say.

The interviews and our analysis were conducted in Chinese. The initial writing was also done in Chinese, and then, a final translation into English was conducted, using direct translations where possible but using paraphrases for difficult parts. This section resulted in a loss of information, and to minimize this, the research team discussed the translation content and made it acceptable to each researcher.

The interviewers converted the recordings into verbatim transcripts after each interview, and IPA was conducted following Smith and colleagues’ (2009) guidelines [28]: (1) reading and rereading; (2) initial noting; (3) developing emergent themes; (4) searching for connections across emergent themes; (5) moving to the next case, (6) looking for patterns across cases.

Each interview was first analysed individually by MM and CYH. After several readings of the transcripts for familiarity, the first emergent themes, which included descriptive, verbal, and conceptual comments, were identified through an initial coding process. These emergent themes were then grouped into higher-order categories, creating a list of superordinate themes for each interview. The research team then reviewed these themes until a consensus was reached and looked for links between the superordinate themes throughout the interviews. The research team then moved on to the next case and finally looked for patterns across cases.

### Ethics

The Ethics Committee of the Wuhan Mental Health Centre approved the study. All potential participants were informed of the purpose of the study and their right to refuse participation without any adverse effect on their support or relationship with the organization and measures to ensure confidentiality. Following this explanation, all individuals agreed to participate in the study and provided written consent to participate.

## Results

### Participant characteristics

The participants were aged between 22 and 55 years, with an average age of 38.5 years; five men and six women took part; the participants were mostly single or never married (63.6%)and lived with their families (63.6%).

The participant’s general information is shown in Table 1.

**Table 1:** Demographic information of the participants

Number	Sex	Age	Illness course(years)	Marital status	Place of residence /co-resident	BPRS cores
A	female	27	10	Married	Home/husband, daughter	23
B	male	35	18	Single	Home/parents	28
C	male	22	5	Single	Home/parents	28
D	male	49	26	Divorced	Home/living alone	25
E	female	51	32	Divorced	Home/living alone	27
F	male	33	8	Married	Home/wife	23
H	female	55	7	Married	Home/husband	23
G	female	24	7	Married	Home/husband, son	21
I	female	49	15	Divorced	Home/living alone	27
J	female	50	10	Divorced	Home/parents	21
K	male	28	9	single	Home/parents	25

Four themes were identified in this study: traumatic illness experiences, influence of the family, motives for recovery, and posttraumatic growth, comprising ten subthemes. (Table 2), each supported by quotes from participants’ records.

**Table 2:** Theme and Subtheme

Theme	Subtheme	Participant endorsement										
		A	B	C	D	E	F	G	H	I	J	K
Traumatic experiences	Symptom- induced distress		x	x	x	x	x	x	x	x	x	
	Stigma and self-stigma	x	x		x	x	x			x	x	x
	Loss of hope, feeling of powerlessness	x	x	x	x	x	x		x	x	x	x
Influence of the family	Staying at home	x	x	x	x	x	x	x			x	x
	Impact on mood	x	x	x	x	x	x	x	x	x	x	x
	Role in medication compliance	x	x			x					x	x
Motives for recovery	For the family						x			x	x	
	Relying on oneself						x		x	x	x	
Post-traumatic growth	Connections with others					x	x	x	x	x	x	
	Individual agency		x	x		x	x			x	x	

### Traumatic illness experiences

Each participant referred to the traumatic experience of having schizophrenia, which included symptom-induced distress, stigma, and feelings of powerlessness.

Symptom-induced distress.

These included both bodily and psychological distress. Even after the individual’s symptoms were under controll, the pain remained fresh in the individual’s mind. The distressed experience might be why the individual continues in treatment or wants to seek help from a doctor.

*“It is so unbearable, worse than death, and people who have never had the illness cannot feel the pain. The onset of the illness is too painful, too torturous. It’s all about the physical discomfort and the pain. The pain in my body is so bad that I can get sick at any time, my chest and back feel like a nail is stuck there; my hands and feet are numb, and it is particularly uncomfortable. “(G)*.

*“It (referring to the symptoms) is not cyclical. It suddenly comes and goes, but wait a bit. The key is not to be anxious; once it happens, your thoughts will not work if you are anxious. You don’t think about anything. I don’t want to think about anything. “(F)*.

*“It’s just hard, hard. I can’t stop thinking about problems. I can’t control them. I don’t want to think about problems. My mind will still think about them. I want to clear my mind, but there are voices in my head that keep talking. It’s hard. I can’t help it. I can’t think about extreme problems, but my head gets dizzy when I think about unnecessary problems.“ (C)*.

*“Couldn’t sleep the next day. My mental state was terrible, and my condition was worse. “(B)*.

### Stigma and self-stigma

Some participants reported being talked about, shunned, isolated, and devalued. They felt lonely, devalued, restricted, and angry. One participant complained that her child was also being bullied. Some participants said they felt low self-esteem because of mental illness, felt pessimistic about the future, avoided contact with the outside world, or feared that others would know about their illness.

*“I walk out. People point at me and murmur: she is the wife of whoever, she is the daughter of whoever. And she has a mental illness. It’s like I’m boxed inside that dungeon.“ “I found that everyone ignored me when I returned from the hospital. They don’t care about me. They teach the children to ignore me. En, I’m so lonely and isolated there. No one wants to care about me.“ “Even my child was bullied. The other kid was bigger and hit my kid on the leg with a big stone. I went to argue, and he ignored me.“ (A)*.

*“The psychological impact of the illness, maybe, is inferiority and a little pessimistic about the future. The inferiority complex means that people with the mental disorders are often looked down upon by others. A person is often looked down upon by others. His life is over. Pessimism means that you feel very pessimistic about your future.“ (B)*.

*“We, as patients, are also stigmatized in society. I was afraid to tell anyone about my illness. But it affected me all my life. Right? I can’t even talk about it. Maybe someday I’ll meet someone I love. I can’t even talk about it. Friends, I lost a lot of friends that I used to have. I initiated contact with them, and they didn’t talk to me. “(J)*.

### Loss of hope, feeling of powerlessness

Most participants described a loss of hope and powerlessness, while others felt scared. Some participants had this feeling for a while after the illness, and some had in this feeling all the time. This feeling was related to being diagnosed with schizophrenia, being on medication for a long time, or having recurrent illness episodes.

*“After I found out my diagnosis was schizophrenia, I felt like I just lost hope in life, I didn’t want to care about anything, I didn’t want to do anything” (F)*.

*“It felt scary, saying something about (the diagnosis of) schizophrenia; it just felt quite scary and could scare people to death. …… When does the second life start? The first life was given to me by my mother; I feel like there is no second life, and I feel like a wasted person when I keep taking medication and eating.“ (L)*.

*“When I got out of the hospital, my ability to survival was poor. I was weak. When I heard the doorbell, I was scared.“ (J)*.

*“It just wasn’t good; I didn’t feel so lucky. Quite a lot of my classmates that I hang out with don’t have it, and I’m the only one who has it.“ (C)*.

*“A bit pessimistic about the future, I guess …… pessimistic means feeling very pessimistic and disappointed about the future.“ “My parents are old, 50 or 60 years old. If they die, how do I do.“ “Schizophrenia, well, can’t be cured completely, mentally very tortured.“ (B)*.

### Influence of the family

Most of the participants lived with their families. Among the three participants (D, E, and I) who did not live with their families, 2 (E and I) also had close contact with their families. Only participant D stated that he rarely communicated with his family. All participants had a permanent home and no experience of homelessness. Most participants stayed at home for some time after being diagnosed with schizophrenia or discharged from the hospital. They reduced their contact with the outside world. As individuals stayed at home, family members interacted intensely with them.

Interactions with family members significantly impacted the participants’ moods and behaviours. Family members’ attitudes and behavior towards the participants’ medication also significantly impacted the participants’ treatment and mood.

### Staying at home

The participants found coping with the stress of relationships and work challenging, so they returned to their homes and had less contact with the outside world. Some participants felt relaxed staying at home, but others experienced diminished capacity and were concerned about their diminished capacity.

*“I do not have a good relationship with strangers and would rather be alone at home with a book and TV. At least I feel more relaxed.“ (K)*.

*“I want to go out to work like everyone else. I cannot do anything if I have this symptom all the time. It’s better not to have those kinds of grumpy people in my cirle. I work in that circle, and if I have that kind of people messing around every, I get a bit unhappy when I face them. I don’t want to see him. He’ll affect my mood.“(C)*.

*“After a while, I was in a bad mood, and my ability to work was weak. Unlike before, I did not want to work for quite a long time. I always stay home, lie in bed, watch TV, and do not want to go out. Then, I returned to the old state of poor performance, that feeling.“(J)*.

### Impact on mood

The attitude of family members towards the individual has a great impact on the individual’s mood. Criticism and blame from family members can cause anger or depression, and worries from family members can increase an individual’s apprehension and depression. When family members are encouraging, understanding, and affirming, the individual will increase communication with family members and will be able to maintain positive behavious.

### Discrimination, blame from family members

The participants experienced impatience, unconcern, and blame from family members, for which the participants felt angry and depressed.

*“My husband, if people ask him who she (meaning A) is. His attitude then becomes like this, too, just saying to ignore her and not to talk to her. I once tried cross-stitching, which requires a lot of patience. When I was halfway through the embroidery, my husband said, ‘What are you embroidering? You can’t even do your housework properly and still embroider this’. He denied me. What housework did I fail to do? Did I not cook, did I not take care of my children, did I not take care of my mother-in-law? “(In an outraged tone) (A)*.

*“The children don’t come to see me either, and I’m particularly depressed and bitter emotionally” (D)*.

*“My parents pick on my sore spot and talk nonsense. En, all this talk is making me feel bad.“(E)*.

### Family worries

The participants experienced a variety of worries from family members, such as family members worrying about their condition, the side effects of medication, the relationships, and the future. Family members’ worries about the individual could add to the individual’s fears.

*“As long as I have this symptom, I can’t do anything. My parents are worried and afraid that my interpersonal relationship outside are shallow. If I want to do something, I need a person to lead me to do it. My parents don’t have anyone right now.“ (C)*.

“*My mother was disappointed in me. She said I would become a farmer like them if I were still so negative. I was a little worried about myself. En, I swallowed the whole bottle of pills. A bottle of clozapine.“ (K)*.

### Family communication, encouragement

The participants experienced that their family members wanted to listen to what was bothering them, cared about them, or were encouraging them and affirming the positive changes they were making. Listening, caring, and support from family members made the participants want to talk and care more about their family members.

*“My parents also say that I’m a different person. They all think I’m good. I’m good to them. (J)*

*“I talk to her (referring to the daughter) a little bit, a little bit (about the condition), sometimes she opens up and tells me to, um, learn to control myself, and she also, she asks me to, but I can’t do it, I told her that too, I said I can’t do it.“ (D)*.

*“My daughter lives where she works, and she’s very concerned about me and often calls me. After all, I was worried about her being a girl, but I have since discovered that she is capable, so I am relieved now.“ (I)*.

### Role in medication compliance

Family members had an important influence on the participants’ medication use. Family members reminded the participants to take their medication. Some family members accompanied the participants to hospital appointments and help with prescriptions, and the participants felt supported. However, some family members made taking medication an essential thing for the participant, constantly reminding the participant and equating failure to take the medication with the onset of the participant’s illness. Other family members force-fed medicines to the participant, and these coercive methods made the participant unhappy and resentful. Some family members were also concerned about the side effects of the medication and asked the participants to stop taking it.

*“I was then always very positive and cooperative in my treatment. At first, it was my 70-year-old father who brought me to the doctor, and then later, I slowly came to the doctor on my own.“ (J)*.

*“My family always asked me if I had eaten or taken my medication. I had to remember to take my medicine and not forget to do so. These were just a few words. I felt like I had accomplished a considerable task.“ (A)*.

*“My mother was afraid I would get sick from my medicine, so she told me not to take it.“ (B)*.

*“Whenever I get angry and don’t take my medicine, my parents think I will be sick.“ (L)*.

*“They (referring to parents) would take a scoop, open my mouth with the scoop, and ask me to take my medicine, and I also felt disgusted.” (E)*.

### Motives for recovery

Individuals wanted to get better, but taking the initiative to take steps to start getting better, rather than avoiding people and situations that made it difficult for them, required a driving force. The individual’s narrative revealed that being for the family and relying on oneself were the motivating factors to increase individual initiative.

### For the family

Individuals took responsibility for their families and wanted to be able to take care of them, such as their elderly parents, younger siblings, and children, to “take on the burden of the family” and to provide financial and emotional care for them. The participants interpreted the improvement of their situation as a way of not “causing trouble” for their families. “easing the burden” on them, and taking responsibility for them.

*“My father is dead too, …. My mother is old, and I have two younger sisters, so I have to bear the burden of my family.“ “If I lose touch with society and drag my family down with me, at the end of the day, it’s all; it’s all hurting myself, it’s all hurting my family.“ (F).*

*“My mum and dad are very old and emaciated…. I want to lighten the burden on my family” “My mum and dad are physically ill. I think this burden I have to pick up. Then I went out to work again, and I forced myself when I was working. Slowly I was able to do the job.“ “Now I can take part of the responsibility of the family, and I also care for my sister, my brother-in-law, my niece, my dad, and my mum.“ (J).*

*“I hope not to give my daughter any trouble. When she needs money, I can help her. The first is not to be hospitalized. I want my life to be about taking medicine, eating, closing my eyes, and not being hospitalized again. When I was in the hospital, those who cared about me, including my parents and my daughter, were affected. I was also sick and had much pain.“(I)*.

### Relying on oneself

Four participants referred to ‘relying on oneself’, which included relying on oneself to manage life’s chores, regulate one’s emotions, take care of oneself, and encourage oneself. Relying on oneself is also an expression of taking responsibility for one’s life and supporting oneself.

*“The reality is that you still have to rely on yourself, you have to do a lot of tedious things in real life by yourself, you can’t be a little bit lazy, it’s like taking care of yourself, if you are a little bit lazy, you will end up not wanting to do it more and more.“ “If you don’t make any progress at all, if you’re not willing to go in a good direction and improve yourself, then the doctor can’t do anything with you, and the medicine can’t do anything with you.“ (I)*.

*“You have to unlock the locks yourself, but if your heart is locked, you can’t open it,“ “You have to rely on yourself, you have to rely on yourself.“ (F)*.

*“We ordinary people, we have to rely on ourselves, …… can’t give up on ourselves.“ (G)*.

*“Now it’s about being strong on your own. Keep yourself in an optimistic frame of mind and look down on some things a little bit.“ (H)*.

### Posttraumatic growth

Some participants reported positive changes associated with their experiences. Posttraumatic growth is the recovery and improvement of physical and mental health from adversity and regaining control over one’s life; posttraumatic growth took time and did not develop linearly. Post-traumatic growth included the subthemes of increased connection with others and individual agency.

### Connections with others

The emotional connection to relationships with others had two components: on the one hand, the individual trusted others, communicated more with them, and felt more supported by them; on the other hand, the individual felt more supported by others.

### Feeling supported by others

Developing trust in others and increased interaction leads to a feeling of support from others. These others were often family members or health professionals, and one participant also talked about relationships with friends.

*“I see how a doctor treats another patient with warmth, the little gestures, the little things taken into consideration, and it touches my heart, and I feel trustworthy. “ (G)*.

*“(I’m) annoyed or unhappy, I feel uncomfortable, but then, well, I talk to the girl.” (E)*.

*“I now talk to my sister when I’m upset about something.” (I)*.

*“I have a friend who knows about my illness and recovery. She’s always been there for me, and she’s very open about it. I cherish this friend.” (J)*.

### Support for others

The participants were more likely to help, had more tolerance for others, and could work with others and share benefits.

*“I am not as aggressive as I used to be, and I can get along well with other people.“, " Because of this illness, no matter how strong people are, there is still a day when they fall. Many things are unexpected. One has to be open-minded, healthy is a must, and living is a victory.“ “In the company, I feel that a team is more powerful than a single person, and I have learned to share now. What I used to have, commission or not, performance or not, I have to take it all into my arms. Now I take some of it out and share it with others.“, “My parents also say I’m better than I used to be. I’m more caring; I used to be very selfish. I used to spend all my money on myself. Now I can take responsibility for my family…Mum and Dad can rely on me. I’m proud of myself now.“ (J)*.

### Individual agency

Individual agency is reflected in how individuals adopt methods to improve their emotions, cope with symptoms, try new behaviours and ultimately empower themselves.

### Emotional self-regulation

The participants used methods to reduce their discomfort and improve their mood when experiencing painful feelings or mental symptoms; they used self-encouragement when it was challenging to continue to persevere in their actions.

*“When I’m upset or unhappy, I feel uncomfortable, but I tell my daughter that I’m not uncomfortable, and sometimes I just go and play with my jumper by myself. It calms me down, so I like to do it” (E)*.

*“If I feel uncomfortable, I’ll walk with my head down for a few minutes or go to bed.“ (C)*.

*“When I’m not happy, I think of something happy, or I go and play cards with my friends.“*

*“It’s still hard to take the trouble to do something for yourself every day and take care of things at home, but it’s better to cheer yourself up and be strong with this. I reassure myself, ‘If I fail, I’ll try again’.“ (F)*.

### Proactive behaviour

The participants took action to try, learn and accomplish things, such as household chores, financial management, and work. Gradual improvement in the ability to do things in action was followed by self-affirmation and increased autonomy. Completing tasks often required constant experimentation and could fluctuate and be repetitive.

*“Sometimes there was supposed to be a price to keep track of when selling things, and (I) didn’t do much of that. Now it’s different, I write down the price sometimes, and I can sell it.“*

*“Now I do more housework; sometimes I clean the house. I wipe the sofa and mop the floor.“ (C)*.

*“Since I have this illness, I can’t say I’ll never do anything for the rest of my life; what if I get old? Then I can only do some simple things and slowly recover that ability. I then went to work overseas for two years.“ (F)*.

*“At home, I bought groceries, started keeping accounts, and basically wrote down everything I bought. I hope not to give my daughter trouble. She needs help when she needs enough money because I used to spend so much money on my daughter. If there is a need for financial help, who can she call? I have to go to help her. I do not need to eat or drink very well in my own life, as I am also gaining weight and cannot eat too well now. That is mainly for my daughter. I do not want to give her trouble.“ (I)*.

*“Once I started working, my ability came back quite OK. I started working and took a few orders, and my boss impressed me. However, after a while, my mood and my ability to work were weaker. Unlike before, I didn’t want to work for quite a long time. I always stayed at home, lying in bed, watching TV, not wanting to go out. I don’t have any orders, and then I’m back to my old, kind of poor state, that kind of feeling. Later, I thought that this would not work, as my mother and father were both ill, and I felt that I had to take up this burden. Then I went back to work, and when I worked, I forced myself to work. Slowly, I was able to do the job. I went from feeling quite overwhelmed at the beginning to getting used to it, and then eventually, I could do it.“ (J)*.

## Discussion

It is evident from the accounts of the individual in this study that the illness causes great suffering to individuals and that after developing schizophrenia, individuals experience or have experienced a lack of hope, a lack of strength, and a lack of ability to face life again in the future.

Previous studies have suggested that developing schizophrenia is a traumatic experience for individuals [29–31]. Some studies have linked traumatic experiences to psychotic symptoms and treatment experiences [32], while others have linked traumatic experiences to shame [33, 34].

People with severe mental illness (SMI) often encounter stigmatizing perceptions of mental illness [35]. These perceptions can lead to social exclusion, discrimination, and microaggressions against people with serious mental illnesses [36–38]. The effects of stigma include self-stigma, where a person internalizes socially stigmatizing messages about mental illness. Self-stigma can lead to depression, low morale, lower self-esteem, poor disease management, social avoidance, and impediments to pursuing and achieving recovery goals [39–42].

Isabella Berardelli suggested that demoralization is a syndrome of existential distress. This symptom may occur in people with chronic mental illness that threatens the integrity of existence or the meaning of people as participants in the world [43]. Frank identified low morale as helplessness, incompetence, declining self-esteem, despair, being stuck in a rut, loneliness, and meaninglessness, possibly followed by a wish to die [44]. Onken argued that the low morale can be a significant obstacle in the recovery process [45]. Ritsher argued that three sub-themes of self-change, pessimism about the future, and feelings of control construct the individual’s sense of powerlessness [46]. A study by Liu Liang and colleagues. on Chinese individuals who had a lived experienced schizophrenia found that individuals lacked clear judgments about their personal experiences in many areas, including physical experiences, mental states, and related factors. The participants often felt nervous, sensitive, and vulnerable in their daily lives and were unsure whether their feelings or judgements were ‘normal’. They lose confidence and become powerless in their lives [47].

However, this suffering comes not only from a sense of stigma and powerlessness but also from the symptoms themselves. The painful experience of symptoms is why individuals seek treatment, and some use hospitalization as the ultimate solution to cope with the pain of their symptoms. Some individuals are opposed to treatment and feel that hospitalization is forced upon them and that prolonged medication increases their sense of powerlessness.

After a traumatic illness experience, individuals often choose to stay at home to reduce the stress on them in terms of relationships and work. As staying in the home becomes more interactive with the family the influence of the family on the individual becomes more apparent.

Many researchers have reported on the effects of family on people with schizophrenia. Individuals who a lived experience of schizophrenia who came from families with high emotional expression (expressing high levels of criticism, hostility, or excessive involvement) have higher relapse rates than those with schizophrenia from families without similar problems [48–50].

Family warmth and positive remarks have been found to have a protective effect and reduce the likelihood of relapse [51].

Johannes Jungbauer and colleagues, in a study of German people diagnosed with schizophrenia, found that at the time of the interview, 41% of the people were still living with their parents or had moved back to their homes [52].

90% of people diagnosed with schizophrenia in China live with their families, compared to 60% in the UK and 40% in the US [53]. Such a high proportion of individuals live with their families and interact more with them, and the influence of family members on individuals is more evident.

This study suggests that an individual’s interaction with family members significantly affects the individual’s mood and behaviour. With family discrimination and blaming, the individual develops negative emotions and impulsive behaviours; he or she may also develop depressive and withdrawal behaviours. Family members’ worries may also increase individuals’ worries about the future and the outside world. At the same time, family members’ willingness to communicate with individuals may also improve their communication with family members strengethening the connection between individuals and their families.

A study by Johannes Jungbauer and colleagues found that re-enforcement of the parent-child relationship may lead to decreased social contact outside the individual’s family [52]. Whereas all of the participants in this study, except D, maintained close relationships with their families. Some participants also had much social contact outside the home. Therefore, the differences between individuals with much social interaction and those with little social contact should be further studied. One possible reason for little social contact outside the home is that family members feel uneasy about the outside world, thus discouraging individuals from social contact with the outside world, For example, in the case of C. A qualitative study by Zhang Yanqing and colleagues in Taiwan also found that when families were not actively involved or supportive of their relatives’ recovery journeys or could not work with their relatives, individuals’ recovery was negatively affected. This study also suggested that families’ overprotection or fear of making changes for their relatives with mental illness prevented people with mental illness from participate in independent learning and decision-making [54].

Because of the prominent influence of family members on individuals, Chinese individuals who experience schizophrenia need to improve their family-individual interactions and change the overprotective response of family members. Family influence was found in this study to manifest in individuals’ motivation to recover.

With medication, the influence of family members is also evident, as individuals are more likely to accept medication if their family members are gently supportive. In contrast, family members ordering or even forcing medication can cause anger in the individual and lead to tension between the family and the individual.

Joanna referred to the primary motivation for recovery as the ‘drive to move forwards’, which is the foundation or starting point for recovery. This forwards momentum includes hope, optimism, determination, belief in a higher power, and an awakening of motivation. In his study, some participants spoke of recovery as a spiritual journey and a connection to a higher power. Finding meaning and purpose is a key part of recovery, and some people seek and find this meaning in their religious beliefs [55].

Janne claimed that religion and spirituality hold a great deal of power in the search for meaning in the lives of people with mental illness [56].

In contrast, the participants in this study did not mention religious beliefs. What, then, constitutes meaning in their lives? According to some participants, “for family” has become the meaning of life. Several participants in this study described that taking responsibility for one’s family was often the turning point in their decision to work towards recovery. Their description suggests that for Chinese participants, family not only has an important influence on them but is also a source of motivation for recovery.

This phenomenon is related to the psychological characteristics of the Chinese people. Yang Guoshu suggests that familism is a major indigenous set of Chinese psychological and behavioural principles and a complex indigenous cultural phenomenon in Chinese society. Familism is the Chinese idea and practice of putting the family first in all matters. Familism aims to maintain the strength and harmony of the family, for which the children must pass on the family line and support and obey their parents. The basis of its ideology is filial piety. The responsibility a child to provide for one’s parents is an important part of familism, and it forms an important part of Chinese life [57]. Eva Yin-han Chung also argued that for Chinese people, identification and connection to family give meaning to life; responsibility and commitment are important factors that motivate people and empower them to live meaningful lives [58].

Abdullah argued that in Asian populations, individuals’ inability to care for their parents when they are old and sick can create a sense of stigma for the individual diagnosed with schizophrenia [16]. In a study by Yin-Ling Irene Wong and colleagues on Chinese individuals diagnosed with schizophrenia and their families, participants with schizophrenia expressed a sense of shame and low self-esteem, and talked about being a burden to their families [53].

This study shows that individuals’ renewed responsibility for parental support, assisting a younger sibling, and raising and helping children is an essential expression of their life’s meaning and catalyses the their recovery. Individuals feel proud if they can achieve these goals. Therefore in the eyes of Chinese individuals, being able to achieve the task of caring for their families gives them a sense of pride, while not being able to do so increases their sense of stigma.

On the other hand, the particpants also express that they are “relying on oneself”, dealing with life’s chores, regulating their emotions, taking responsibility for their lives, and supporting themselves.

Nonetheless, the participants also stated that they wanted to be “relying on oneself” a concept that requires self-support and motivates individuals to take action to deal with life’s chores, regulate their emotions, and take responsibility for their own lives to achieve self-support.

The factors suggestive of traumatic growth in this study are the connection with others and the individual’s agency. The other connections mentioned more often by individuals were relationships with family members.

“For the family” and “relying on oneself” are cognitive demands, while individual agency is a behavioural response. Guided by the concepts of “for the family” and “relying on oneself " individuals adopt proactive behaviours directed towards helping the family, thus strengthening the individual’s bond with the family.

Several researchers have described recovery as a transformative process of self-discovery and self-renewal, which involves adjusting one’s attitudes, feelings, perceptions, beliefs, roles, and life goals [12, 58, 59]. Yulia and colleagues’ study considered the individual’s sustained efforts towards positive transformation and improvement as the basis of the recovery process. The opposite of this is abandonment, i.e., the acceptance of the individual’s negative identity as an individual with a chronic illness and the lack of intrinsic motivation to want to get better [60]. Larry Davidson claimed that rebuilding an “enhanced sense of self” protects people from being struck down by illness and provides a solid foundation for their recovery [49]. Onken argued that rejuvenation is often rooted in agency and self-activity [45]. A study by Deegan and colleagues identified the right to individual choice and empowerment as important elements of recovery [61]. Markowitz suggested that for individuals to recover from the trauma of schizophrenia, the healing process involves not only a new lifestyle and control of symptoms but also increasing proficiency in overcoming stigma and discriminatory experiences in the social sphere [62].

It is an important direction of recovery to promote the development of self-discovery and the self-ability of the individual.

It is worth noting that some participants in this study took good care of themselves as an important way to relieve their families’ burden and treat them well. They reported if they are not well, their families will suffer; if they are well, they can ease the burden on their families. Thus, “relying on oneself” is associated with “for one’s family”.

It is clear from this study that both the self and the family are emphasized in the individual’s experience of recovery. The individual is an individual in the family. The honour and shame of the individual are closely linked to the honour and shame of the family. Therefore, the individual’s efforts can improve the family’s situation. So the individual’s efforts are as much for himself as for his family.

### Limitations

The study may be limited for several reasons. The participants had certain geographical limitations. The study was carried out in only one large city in China. Our sampling method may have resulted in selection and response bias. The participants were recruited through clinical staff. In addition to recommending individuals who fit the study’s inclusion criteria, clinical staff tended to refer people with good relationships.

## Conclusion

People with schizophrenia living in China have undergone significant traumatic experiences and have profound interactions with their families. Posttraumatic growth enables an increase in the individual’s connection to others and autonomy. The study also found that individuals did not receive adequate support outside their families. These findings suggest that the impact of individual autonomy and family relationships should be considered in services that promote recovery and that support outside the home should be enhanced.

Careful consideration of the impact of Chinese culture on individuals and the establishment of recovery in a Chinese cultural context is an important issue in Chinese psychiatric recovery services.

## Electronic supplementary material

Below is the link to the electronic supplementary material.

Supplementary Material 1 Timetable

## Data Availability

The datasets generated and analysed during the current study are not publicly available due [ this is a Qualitative Study ] but are available from the corresponding author on reasonable request.
