# Supplementary material for: Recovery journey of people with a lived experience of schizophrenia: a qualitative study of experiences
Source: BMC Psychiatry. 2023 Jun 27;23:468. doi: 10.1186/s12888-023-04862-1 (PMC10294500; doi:10.1186/s12888-023-04862-1)
Supplement: Supplementary file 1 — Supplementary Material 1 Timetable [file 12888_2023_4862_MOESM1_ESM.docx]

Timetable

1 Can you tell me about your experience of being ill? How does the condition change?

2 What can you tell me about the impact of schizophrenia on your life?

3 Can you tell me how you feel about your schizophrenia?

4 Can you tell me how schizophrenia has affected your relationships with your family? How have you coped?

5 Can you tell me how schizophrenia has affected your relationships with other people? How have you coped?

6 Can you tell me about your experiences with treatment for schizophrenia? How do you feel about these experiences?

7 Can you tell me how you see recovery from your illness?

8 Ask the patient to describe the current life situation, including work, interpersonal communication, family relationships, and religious beliefs. Did something make a recovery better? Did anything make a recovery worse? How do you feel about these changes?
